# The Making of the Modern Practitioner

**Published:** 1911-11-25

**Authors:** 


### THE MAKING OF THE MODERN PRACTITIONER.

### The Internal Secretions in Therapeutics.

Some years ago a short volume entitled "Minor Maladies" proved its author, Dr. Leonard Williams, to be an uncommonly shrewd practical clinician of a type not any too numerous among consulting physicians. The general practitioner speedily discovered the value of this book, and added it to his book-shelves; for it supplied him with most useful assistance in dealing with the problems of those minor medical diseases which constitute the great bulk of ordinary general practice. Not only so; it also showed that rule-of-thumb methods are as useless in these maladies—which, because they are "minor" in pathological significance, are not by any means necessarily simple to treat and cure—as in more serious conditions; and that intelligent thinking is just as essential in dealing with a common cold or sore throat as in treating typhoid fever or diabetic coma.

Since the publication of the book above referred to, its author has been mainly concerned with the therapeutics of the ductless glands, and indeed of all those glands which are believed to possess an internal secretion. In a recent paper * he summarises his present convictions as to future progress along these lines. To the general practitioner the matter is one of importance, since many of the diseases which Dr. Williams proposes to deal with are those which are of frequent occurrence and are often very troublesome to treat. The general practitioner has long ago learned the value of thyroid extract, for instance, in definite cases of myxœdema. But Dr. Williams extends the sphere of action of this glandular extract very much further than myxœdema. Nocturnal enuresis, chorea, and rheumatism are among the manifestations attributed to subthyroidism, and to be treated accordingly.

Before examining in detail a certain highly ingenious train of reasoning which the author puts forward, it must be premised that for nocturnal enuresis the general experience is that thyroid extract does little good, and that Dr. Williams' enthusiasm for it is not borne out in the practice of those who, on his recommendation, have given it a trial. For the treatment of chorea, rheumatism, and rheumatic nodules, experience has still to accumulate as to the virtues of thyroid extract.

Dr. Williams starts with the proposition that cod-liver oil is valuable in the prophylaxis and treatment of tuberculosis, and that it is superior to any other oil, animal or mineral. He further points out that the more refined the oil, the less efficacious it is found to be; whereas dried extract of cod's liver is almost as active as the crude oil. Hence is postulated the existence of some internal hepatic secretion of the cod which is beneficial to human beings.

The next step is to connect the physical signs of tuberculosis with those of Graves' disease. The freely acting skin, the tendency to hirsuties, the keen intelligence, the optimism, the dyspepsias, the tachycardia: all these are noted in support of the resemblance. Further, these two conditions are both made worse by thyroid extract, and better by cod-liver oil. Tubercle, it is argued, does not as a rule attack subthyroidic people such as the cretinoid, obese and gouty, whereas it is peculiarly liable to attack families some members of which exhibit hyperthyroidism; thus it is suggested that the liability to tubercle is a function of hyperthyroidism.

How then is such baneful hyperthyroidism to be controlled? Connecting the vast preponderance of females amongst the sufferers from Graves' disease with disorders of the generative organs, ovarian inadequacy was thought to be possibly at the back of hyperthyroidism; but trial showed that no satisfactory results could be obtained on these lines. Then pituitrin was in turn experimented with, because it stimulates ovarian activity, with equal want of success. Lastly, certain analogies between myxœdema and jaundice were connected with the value of the liver of codfish in tuberculosis. Thus slow pulse, subnormal temperature, and physical lethargy are characters both of icteric and of myxœdematous persons; indeed, the latter are not infrequently faintly jaundiced. This somewhat roundabout argument suggested that an endeavour should be made to render the blood of patients suffering from Graves' disease like that of those suffering from jaundice, by administering bile salts intramuscularly. Paratoxin, a French preparation, has been used thus with highly encouraging results.

The conclusion which Dr. Williams would have us draw is that thyroid excess is a function of biliary inadequacy, and that this explains the virtue of cod-liver oil in the treatment of tuberculosis, itself a result of hyperthyroidism. The chain of argument is interesting, though it would be easy to show that it has serious flaws. Subthyroidism, on the other hand, is held to be an important factor in acute rheumatism, chorea, subcutaneous nodules, tonsillitis, and erythema nodosum, all of which are described as allotropic manifestations of one disease. In the prophylaxis of all forms of this "alloptropic disease," one-tenth of a grain of thyroid extract is advised for adults, to be taken three times a day; for a therapeutic dose a quarter of a grain is enough to start with, increased later if necessary. The usual large doses of this extract are condemned, except for actual myxœdema.

Whether these curious speculations will stand the test of experience time will show. Certainly the therapeutic promise of the internal secretions is such that great developments may eventually be expected along these lines. But until knowledge of these secretions is much more advanced than it now is, it is impossible to do more than blindly grope our way; and empiricism must at present be our chief guide to the usages of this new therapeusis.

* *Practitioner*, November 1911.

